# A multidimensional gender analysis of health technology self-efficacy among people with Parkinson’s disease

**DOI:** 10.1007/s00415-024-12635-3

**Published:** 2024-08-22

**Authors:** Irene Göttgens, Sirwan K. L. Darweesh, Bastiaan R. Bloem, Sabine Oertelt-Prigione

**Affiliations:** 1 Research Institute for Medical Innovation, Department of Primary and Community Care, Radboud University Medical Center, Nijmegen, The Netherlands; 2 Donders Institute for Brain, Cognition and Behavior, Center of Expertise for Parkinson & Movement Disorders, Department of Neurology, Radboud University Medical Center, Nijmegen, The Netherlands; 3 AG 10 Sex- and Gender-Sensitive Medicine, Medical Faculty OWL, University of Bielefeld, Bielefeld, Germany

**Keywords:** Digital health technologies, Parkinson’s disease, Gender, Intersectional analysis, Health technology self-efficacy, Health technology attitude

## Abstract

**Background:**

Digital health technologies (DHT) enable self-tracking of bio-behavioral states and pharmacotherapy outcomes in various diseases. However, the role of gender, encompassing social roles, expectations, and relations, is often overlooked in their adoption and use. This study addresses this issue for persons with Parkinson’s disease (PD), where DHT hold promise for remote evaluations.

**Methods:**

We conducted a cross-sectional survey study in the Netherlands, assessing the impact of gender identity, roles, and relations on health technology self-efficacy (HTSE) and attitude (HTA). An intersectional gender analysis was applied to explore how gender intersects with education, employment, disease duration, and severity in influencing HTSE and HTA.

**Results:**

Among 313 participants (40% women), no significant correlation was found between gender identity or relations and HTSE or HTA. However, individuals with an androgynous (non-binary) gender role orientation demonstrated better HTSE and HTA. The exploratory intersectional analysis suggested that sociodemographic and clinical factors might affect the influence of gender role orientations on HTSE and HTA, indicating complex and nuanced interactions.

**Conclusion:**

This study highlights the importance of investigating gender as a multidimensional variable in PD research on health technology adoption and use. Considering gender as a behavioral construct, such as through gender roles and norms, shows more significant associations with HTSE and HTA, although effect sized were generally small. The impact of gender dimensions on these outcomes can be compounded by intersecting social and disease-specific factors. Future studies should consider multiple gender dimensions and intersecting factors to fully understand their combined effects on technology uptake and use among people with PD.

**Supplementary Information:**

The online version contains supplementary material available at 10.1007/s00415-024-12635-3.

## Introduction

In recent years, digital health technologies (DHT) have revolutionized the self-tracking of bio-behavioral states and the assessment of pharmacological therapy outcomes in numerous diseases. Particularly for individuals living with Parkinson’s disease (PD), a condition characterized by its multifaceted as well as progressive nature, these technologies harbor the potential to greatly enhance quality of life through meticulous symptom monitoring and informed self-management of medication and lifestyle choices [1–4].

Despite the growing interest in the potential of technology to optimize disease management and support remote assessments in the setting of clinical trials, a gender-sensitive analysis is rarely incorporated. A gender sensitivity analysis explores how distinct gender dimensions (Box 1), such as gender identity, gender roles and relations, impact the design and implementation of health technologies and affect their uptake and use [5, 6].

**Box 1:** Gender dimensions and descriptions

Gender dimensions	Description
Gender identity	Refers to and individual’s sense of self (i.e., identifying as a woman, man, non-binary person etc.)
Gender roles and norms	Refer to social expectations associated with being a man, woman or non-binary person in a given society (i.e., societal structures that lead to shared ideas about what constitute e.g., masculinity, femininity and androgyny, and how these concepts are socially (in)congruent with certain gender identities)
Gender relations	Refers to ways in which power, resources and responsibilities are distributed between people in a given society and/or household (the impact of gender on e.g., responsibilities and task division in couple relationships) based on gender norms

Several studies found that men generally report higher technology self-efficacy than women, which is considered a key motivation construct underpinning health technology use [7–9]. However, recent reviews in the STEM field concluded that identity-level explanations concerning women’s abilities, interest or self-efficacy toward (health) technology are insufficient for explaining these gender gaps [9, 10]. The authors called for more in-depth investigations into stereotypical gender roles and norms that can act as cultural barriers for exposure and access to technology that hinder self-efficacy perceptions. For example, Huffman et al. (2013) found that masculine gender roles seem more predictive of higher technology self-efficacy than sex assigned at birth or gender identity [11]. These findings are in line with stereotype threat theory concerning how people react to and interact with (health) technology according to culturally desired gender roles *related* to their gender identity, rather than the identities themselves [12].

While several studies in the field of PD and DHT have focused on the impact of digital health interventions on general self-efficacy of people with PD [13, 14], to date none have investigated health technology self-efficacy (HTSE) as a specific predictor for the uptake and use of digital health interventions, let alone, applied a gender-sensitive perspective. A recent study regarding the use of health technologies among elderly patient populations in the Netherlands observed that men had more intention to use mHealth applications compared to women [15]. The authors suggest that “traditional ways of thinking about social role division with regard to technology use should be disregarded” (e.g., stereotypical beliefs such as ‘men are more tech-savvy than women’) [15, 16]. To increase health technology uptake among elderly women, focus should be placed on the technology adoption factors that best define patterns of differences between men and women in accessibility, digital literacy, and intention to use, and their identification should be part of a participatory design process of health technologies [17, 18]. Furthermore, to help tailor digital health interventions more effectively to specific subgroups of people with PD, assessing intersections between different gender dimensions with other social and clinical variables and their compounded impact on HTSE can be used to further understand and explore within-group differences among men and women with PD and their intention to use DHT [19].

To address this knowledge gap, our study ventures beyond the traditional scope, championing a multidimensional gender analysis to investigate its impact on HTSE of men and women with PD in the Netherlands. Building on previous studies, we hypothesized that individuals with androgynous gender roles would exhibit better HTSE and attitudes toward health technology [20–22]. In a secondary exploratory analysis, we showcase the application of an intersectional gender analysis and examine the nuanced ways in which gender dimensions intersect with education type, employment status, disease duration and disease severity to steer HTSE and attitudes toward health technology.

## Materials and methods

### Design

This cross-sectional study was embedded within the Proactive and Integrated Management and Empowerment in Parkinson’s Disease—Netherlands (PRIME-NL) study, a prospective cohort study of persons with Parkinsonism and their caregivers [23]. The current study focused on men and women with PD who completed the questionnaire on gender dimensions and health technology self-efficacy and were recruited between March 2020 and March 2021. The PRIME-NL study was reviewed by the Ethical Board of the Radboud University Medical Center [file 2019–5618]. All participants signed a digital informed consent before inclusion in the study.

### Study population

Participants were eligible for this study if they met the following criteria: diagnosed with Parkinson’s Disease or Parkinsonism; 18 years of age or older; able to read and understand Dutch; willing and able to complete an online survey; providing digital written informed consent.

### Determinants

#### Outcome measure

##### Health technology self-efficacy & attitude

**Fig. 1:**
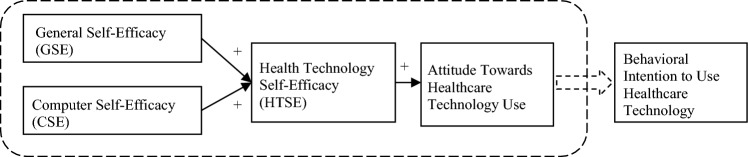
Conceptual model for people’s intention toward healthcare technology uptake, Rahman et al. (2016)

We used the health technology self-efficacy (HTSE) questionnaire developed by Rahman et al. (2016). Rahman defines health technologies as: “Any devices that are used to diagnose, monitor, or treat health or any medical conditions associated with an individual’s health” [7]. Figure 1 presents the conceptual model on which the HTSE questionnaire was built.

The survey assesses *general self-efficacy* (GSE, 7 statements related to individuals’ perceptions in a general contexts about their ability to cope with different situations and various challenging environments, which are similar items to GSE scales previously used in PD research [24]), *computer self-efficacy* (CSE, 6 statements related to the individuals’ perceptions about their ability to use a computer) and *health technology self-efficacy* (HTSE)-specific factors related to (1) the use of healthcare technology in general (4 statements), (2) receiving health services that make use of healthcare technology (4 statements) and (3) using internet health services (4 statements). Lastly, it includes 5 statements to measure general *healthcare technology attitude* (HTA) of the participants toward the use of healthcare technology. Participants were asked to score each of the statements on a 7-point Likert scale from 1 (strongly disagree) to 7 (strongly agree). The sum score was calculated as the mean overall score. Higher scores on the GSE, CSE, and HTSE represent more positive self-reported perceptions of an individual’s ability to cope with challenging situations and computer and health technology use. Higher scores on HTA indicate a more positive attitude toward using health technology to monitor health.

#### Gender dimensions assessments

To capture multiple gender dimensions in our survey, we used a previously developed gender assessment that include state-of-the-art options for operationalizing the gender dimension applied in this study: *Gender identity*, *gender roles* and *gender relations* [20] (Supplement 1 1).

#### Gender identity

The dimension of gender identity refers to the gendered sense of self of a person and is operationalized through *self-reported gender identity*. Self-reported gender identity refers to the self-identification of a person—the response options were woman/man/non-binary or a ‘none of the above’ with an open text option.

#### Gender roles

Gender roles refer to stereotypical behaviors, roles and attitudes that are defined, in a specific cultural context, as more appropriate or desirable for men or women. Gender roles were operationalized through gender role orientation; a person’s orientation toward personality traits that are culturally associated with desirable masculine and feminine behaviors. The 60-item Bem Sex Role Inventory (BSRI) was used to assess people’s perception of their psychological gender role orientation. The instrument uses a 7-point Likert scale ranging from 1 (never or almost never true) to 7 (always or almost always true) for stereotypical masculine (*n* = 20; e.g., ambitious, dominant) and feminine (*n* = 20; e.g., affectionate, gentle) descriptors, and neutral filler items (*n* = 20; e.g., sincere, conscientious). Individuals with an androgynous self-concept score high (>4.9) on both the masculine and feminine scales, whereas undifferentiated individuals score low (≤4.9) on both these scales. People with strong masculine or feminine self-concepts score high on only one of these scales. Women who score high on only the feminine scale and men who score high on the only masculine scale are considered having a gender role orientation congruent with their gender identity, whereas women who score high on only the masculine scale and men who score high on only the feminine scale are considered to have a gender role orientation crossed with their gender identity [24].

#### Gender relations

Gender relations define how people, according to cultural context, interact with others and how others relate to them, depending on their attributed sex assigned at birth or perceived gender identity [25]. We operationalized gender relations in the private domain through living situations (married or living with/without a partner), division of household labor and relative income. Participants rated their housework responsibilities on 7 core tasks (cooking meals, cleaning, laundry, maintenance and repairs, etc.) regarding “whom in the household usually does the household task”. Response options were someone else (0), spouse/partner (1), shared equally (2), respondent (3). Mean scores were calculated with higher scores indicating increased participant involvement in household labor. Relative income was assessed by asking participants about their proportional earning in their household. Relative income was categorized ranging from 0 to 100%.

### Statistical analysis

Linear regression models were fitted with individual GSE, CSE, HTSE and HTA sum scores as outcome and gender identity, gender role orientation, household labor division score and relative income as determinants. The sum scores were included as a continuous variable and models were adjusted for age and education level. Living situation was used as a determinant for gender relations. Therefore, only participants that indicated that they were married/living with a partner were included in the analyses related to gender relations. A multiplicity adjusted *P* value <0.0127, calculated using the Family-Wise Error (FWE) threshold to adjust for multiple analyses, indicated statistical significance for the GSE, CSE, HTSE sum score, and HTA score [26].

Additionally, an exploratory intersectional gender analysis was performed. The data were disaggregated creating 8 separate datasets, one for each of the selected social and clinical variables; (1) low/medium education level and (2) higher education level, (3) employed and (4) unemployed, (5) less than 6 years of clinical disease duration and (6) more than 6 years of clinical disease duration and (7) unilateral or bilateral involvement disease disability (Stage 1–2) and (8) mild to moderate bilateral, severe or wheelchair bound disease disability (Stage 3–5). These social and clinical variables were selected based on their potential influence on access, understanding and use of health technologies in people with PD and their availability in our dataset [4, 27]. The intersectional gender model for each dataset was fitted with the HTSE and HTA sum scores as outcome and gender identity, gender role orientation, household labor division score and relative income as determinants. Statistical analyses were performed using R Studio Version 1.1.463.

## Results

### Population characteristics

Table 1 shows the clinical and demographic characteristics of the study population. In total, 313 people with PD completed our survey, of which 126 (40%) were women. The mean age was 67.2 ± 8.2 years, with women being younger (65.3 ± 8.4, *P* < 0.001), having a longer clinical disease duration (6.4 ± 4.6, *P* < 0.001) and living more often alone (26%) or in facilitated care (2.4%) compared to men (*P* = 0.004).

**Table 1:** Clinical and demographic characteristics of the study population overall and by gender identity

	Overall (*n* = 313)	Men (*n* = 187)	Women (*n* = 126)
Diagnosis (*n* (%))			
Parkinson’s disease	295 (94)	174 (93)	121 (96)
Parkinsonism	18 (6)	13 (7)	5 (4)
Age* (mean (SD) in years)	67.6 (8.2)	69.0 (7.7)	65.6 (8.6)
Disease duration* (mean (SD) in years)	5.7 (4.7)	5.1 (4.6)	6.5 (4.6)
Hoehn & Yahr score (*n* (%))			
Stage 1: Unilateral involvement only	86 (28)	49 (27)	37 (31)
Stage 2: Bilateral involvement	109 (36)	71 (39)	38 (31)
Stage 3: Mild to moderate bilateral disease	61 (20)	41 (22)	20 (17)
Stage 4: Severe disability	40 (13)	19 (10)	21 (17)
Stage 5: Wheelchair bound	8 (2.6)	3 (1.6)	5 (4.1)
Unknown	9	4	5
Ethnicity (*n* (%))			
Dutch	310 (99)	186 (99)	124 (98)
Other	0 (0)	0 (0)	0 (0)
Unknown	3 (1)	1 (1)	2 (2)
Education level (*n* (%))			
Low education	59 (19)	32 (17)	27 (21)
Medium education	87 (28)	51 (27)	36 (29)
Higher education	165 (53)	103 (55)	62 (49)
Unknown	2 (0.6)	1 (0.5)	1 (0.8)
Living situation* (*n* (%))			
Alone	59 (19)	26 (14)	22 (26)
With partner or family	250 (80)	160 (86)	90 (71)
Facilitated care	4 (1.3)	1 (0.5)	3 (2.4)
Working status			
Working	65 (21)	37 (20)	28 (22)
Not working	248 (79)	150 (80)	98 (78)

* Significant group differences reported

Table 2 shows the gender dimensions and the overall health technology self-efficacy scores of the study population. Most participants favored an undifferentiated (35%) or a feminine (31%) gender role orientation. Men and women differed significantly in their gender role orientations (*P* < 0.001) with more gender role congruent women (feminine) compared to men (masculine). On the dimension of gender relations, women reported to be more engaged in household labor (*P* < 0.001) and having lower relative income (*P* < 0.001) compared to men in our sample. The mean overall health technology self-efficacy (5.0 ± 1.2) and health technology attitude (5.3 ± 0.9) scores showed no statistical difference between men and women with PD.

**Table 2:** Gender dimensions and health technology scores of the study population

	Overall (*n* = 313)	Men (*n* = 187)	Women (*n* = 126)
Gender role orientation* (*n* (%))			
Androgynous	49 (16)	35 (19)	14 (11)
Feminine	96 (31)	33 (18)	63 (50)
Masculine	58 (19)	49 (26)	9 (7.1)
Undifferentiated	110 (35)	70 (37)	40 (32)
Gender relations*			
Household labor score (mean (SD))^a^	13.4 (3.6)	12.6 (3.1)	14.7 (3.9)
Relative income (*n* (%))^b^			
0–25%	32 (12)	3 (1.7)	29 (29)
26–50%	52 (19)	30 (17)	22 (22)
51–75%	70 (25)	49 (28)	21 (21)
76–100%	121 (44)	94 (53)	27 (27)
Unknown	38	11	27
General self-efficacy score (mean (SD))	4.9 (1.0)	4.9 (1.0)	4.8 (1.0)
Computer Self-Efficacy Score (mean (SD))	4.7 (1.4)	4.8 (1.3)	4.6 (1.5)
Overall health technology self-efficacy score (mean (SD))	5.0 (1.2)	5.0 (1.2)	5.0 (1.1)
General health technology self-efficacy	5.0 (1.2)	5.0 (1.2)	5.0 (1.2)
Receiving health technology services	5.1 (1.2)	5.1 (1.2)	5.0 (1.2)
Using internet health services	4.9 (1.3)	4.9 (1.3)	5.0 (1.3)
Health Technology Attitude (mean (SD))	5.3 (0.9)	5.3 (0.8)	5.3 (0.9)

* Significant group differences reported

^a^Higher household labor scores reflect increased participants involvement in household labor compared to their partner

^b^Participants’ self-reported relative household income as compared to their partner/spouse

### Associations between gender dimensions and health technology self-efficacy dimensions

The results of our model indicate a nominally significant difference in computer self-efficacy (CSE) between women and men, with women scoring slightly lower. Additionally, individuals with an androgynous gender role orientation showed a notably higher general self-efficacy (GSE), with an effect size of 0.55 (SD = 0.15), which was statistically significant (*P* < 0.0127). Additionally, their health technology self-efficacy (HTSE) was nominally higher with an effect size of 0.42 (SD = 0.17), showing significance (*P* = 0.0127 − 0.050). Furthermore, they exhibited a more favorable health technology attitude (HTA) with an effect size of 0.38 (SD = 0.13), also significant (*P* < 0.0127). Compared to individuals with other gender role orientations, those with an androgynous orientation demonstrate consistently higher efficacy and positive attitudes toward health technology.

Conversely, having an undifferentiated gender role orientation was significantly associated with poorer outcomes in terms of GSE, overall HTSE, and HTA. Specifically, the effect size for GSE was −0.46 (SD = 0.11), indicating a statistically significant decrease (*P* < 0.0127). Similarly, the effect size for overall HTSE was −0.32 (SD = 0.13), also significant (*P* = 0.0127 − 0.050), and for HTA, the effect size was −0.33 (SD = 0.10), significant (*P* < 0.0127) (Table 3). No significant associations were found between the dimensions of gender identity and gender relations and HTSE and HTA.

**Table 3:** Associations between gender dimensions and General, Computer and health technology self-efficacy and attitude

Gender identity	General *β* (SD)	Computer *β* (SD)	Health technology *β* (SD)				Attitude *β* (SD)
	GSE	CSE	HTSE SUM	HTSE General	HTSE Receiving	HTSE Using	ATT
Women	−0.16 (0.12)	−0.35 (0.16)*	−0.11 (0.13)	−0.09 (0.14)	−0.20 (0.14)	−0.03 (0.15)	−0.04 (0.10)
Gender role orientation							
Androgynous	0.55 (0.15)**	0.18 (0.21)	0.42 (0.17)*	0.52 (0.18)	0.37 (0.18)*	0.37 (0.19)	0.38 (0.13)**
Feminine	−0.32 (0.12)**	−0.29 (0.17)	0.08 (0.14)	0.03 (0.15)	0.09 (0.14)	0.10 (0.16)	0.10 (0.10)
Masculine	0.66 (0.14)**	0.33 (0.20)	0.01 (0.16)	−0.16 (0.17)	0.01 (0.17)	0.16 (0.18)	0.01 (0.13)
Undifferentiated	−0.46 (0.11)**	−0.06 (0.16)	−0.32 (0.13)*	−0.23 (0.14)	−0.31 (0.14)*	−0.42 (0.15)**	−0.33 (0.10)**
Gender relations							
Household labor score	0.05 (0.02)*	0.04 (0.03)	0.03 (0.02)	0.00 (0.02)	0.03 (0.02)	0.05 (0.03)*	0.02 (0.02)
Relative income	0.04 (0.06)	0.06 (0.09)	−0.03 (0.07)	−0.07 (0.07)	0.00 (0.07)	−0.01 (0.08)	−0.11 (0.05)

Gender identity category ‘non-binary’ is excluded from the table due to the absence of data in this category

For each independent variable, the analysis was adjusted for age and education level. *β* coefficients are presented for gender role orientation as compared to the other categories (category (1)—reference groups (0)) and for Relative Income as continuous predictor with steps of 25% increase in relative income

*P* value: * = 0.0127 − 0.050; ** <0.0127

### Secondary intersectional analyses: associations between gender dimensions, education, employment status, clinical disease duration, disease severity and health technology self-efficacy and health technology attitude

Our initial exploratory intersectional gender model showed that individuals with an androgynous gender role orientation, particularly those who were higher educated (*β* = 0.46, SD = 0.20), unemployed (*β* = 0.44, SD = 0.20), or experiencing unilateral to mild disease severity (H&Y stage 1–2) (*β* = 0.52, SD = 0.22), demonstrated a nominally better overall HTSE (Table 4). Individuals with an undifferentiated gender role orientation exhibited poorer outcomes on the HTSE scale, a trend that was particularly observed in higher educated (*β* = −0.46, SD = 0.17), unemployed individuals (*β* = −0.35, SD = 0.15), or those with a clinical disease duration exceeding 6 years (*β* = −0.52, SD = 0.21). We did not identify significant associations when analyzing the intersection of gender identity and gender relations with the selected social and clinical demographics.

**Table 4:** Secondary exploratory intersectional analyses: association between gender dimensions, education, employment status, disease duration, disease severity and health technology self-efficacy

Gender identity	Health technology self-efficacy							
	Health technology self-efficacy sum score (*β* (SD))							
	Low/med educated	Higher educated	Employed	Un-employed	<6 years disease duration	>6 years disease duration	Hoehn & Yahr score < 3	Hoehn & Yahr score > 3
Woman	−0.05 (0.19)	−0.13 (0.17)	−0.26 (0.24)	−0.07 (0.15)	−0.23 (0.18)	−0.03 (0.23)	−0.31 (0.16)	0.22 (0.23)
Gender role orientation								
Androgynous	0.18 (0.29)	0.46 (0.20)*	0.40 (0.29)	0.44 (0.20)*	0.41 (0.21)	0.44 (0.33)	0.52 (0.22)*	0.31 (0.30)
Feminine	0.15 (0.19)	0.02 (0.19)	0.25 |(0.27)	0.01 (0.16)	−0.26 (0.18)	0.38 (0.22)	−0.05 (0.17)	0.13 (0.25)
Masculine	−0.42 (0.29)	0.11 (0.19)	−0.37 (0.25)	0.16 (0.20)	0.05 (0.19)	−0.06 (0.32)	0.04 (0.19)	0.08 (0.31)
Undifferentiated	−0.06 (0.19)	−0.46 (0.17)**	−0.16 (0.26)	−0.35 (0.15)*	−0.08 (0.18)	−0.52 (0.21)*	−0.27 (0.17)	−0.34 (0.23)
Gender relations								
Household labor score	0,05 (0.04)	0.04 (0.03)	−0.01 (0.05)	0.05 (0.03)	0.07 (0.03)	0.07 (0.04)	−0.01 (0.03)	0.06 (0.05)
Relative income	−0.02 (0.11)	−0.04 (0.09)	−0.02 (0.14)	0.01 (0.08)	0.09 (0.10)	0.01 (0.13)	−0.04 (0.09)	0.04 (0.13)

Gender identity category ‘non-binary’ is excluded from the table due to the absence of data in this category

For each independent variable, the analysis was adjusted for age. *β* coefficients are presented for gender role orientation as compared to the other categories (category (1)—reference groups (0)) and for Relative Income as continuous predictor with steps of 25% relative income increase

*P* value: * <0.050; ** <0.010

In our second exploratory intersectional gender model, we found a nominally better HTA associated with androgynous individuals who were higher educated (*β* = 0.40, SD = 0.18), unemployed (*β* = 0.36, SD = 0.15), or had a clinical disease duration of less than 6 years (*β* = 0.41, SD = 0.17) (Table 5). On the contrary, individuals favoring an undifferentiated gender role orientation and who were lower educated (*β* = −0.32, SD = 0.14), unemployed (*β* = −0.38, SD = 0.11), had a shorter disease duration (*β* = −0.38, SD = 0.14), or higher disease severity (H&Y stage 3 or higher) (*β* = −0.46, SD = 0.17) had worse outcomes on the HTA scale. No significant associations were found between the intersections of gender identity or gender relations and education level, employment status, clinical disease duration, or disease severity.

**Table 5:** Secondary exploratory intersectional analyses: association between gender dimensions, education, employment status, disease duration, disease severity and health technology attitude

Gender identity	Health technology attitude Attitude toward Health Technology (*β* (SD))							
	Low/med educated	Higher educated	Employed	Unemployed	<6 years disease duration	>6 years disease duration	Hoehn & Yahr score < 3	Hoehn & Yahr score > 3
Woman	−0.01 (0.14)	−0.02 (0.14)	−0.21 (0.21)	0.01 (0.12)	−0.01 (0.15)	−0.04 (0.16)	−0.04 (0.13)	−0.04 (0.18)
Gender role orientation								
Androgynous	0.25 (0.21)	0.40 (0.18)*	0.49 (0.25)	0.36 (0.15)*	0.41 (0.17)*	0.33 (0.23)	0.31 (0.17)	0.45 (0.23)
Feminine	0.33 (0.14)*	−0.10 (0.16)	0.07 (0.23)	0.12 (0.12)	0.07 (0.15)	0.22 (0.16)	−0.04 (0.13)	0.28 (0.19)
Masculine	−0.30 (0.22)	0.07 (0.16)	−0.32 (0.22)	0.13 (0.15)	0.04 (0.16)	−0.12 (0.23)	0.08 (0.15)	−0.08 (0.24)
Undifferentiated	−0.32 (0.14)*	−0.27 (0.15)	−0.10 (0.23)	−0.38 (0.11)***	−0.38 (0.14)**	−0.30 (0.15)	−0.20 (0.13)	−0.46 (0.17)**
Gender relations								
Household labor score	0.03 (0.02)	0.02 (0.02)	−0.02 (0.03)	0.02 (0.02)	0.01 (0.02)	−0.00 (0.02)	0.01 (0.02)	0.02 (0.03)
Relative income	−0.13 (0.08)	−0.09 (0.07)	−0.03 (0.11)	−0.04 (0.06)	−0.07 (0.07)	0.02 (0.08)	−0.10 (0.06)	0.03 (0.08)

Gender identity category ‘non-binary’ is excluded from the table due to the absence of data in this category

For each independent variable, the analysis was adjusted for age. *β* coefficients are presented for gender role orientation as compared to the other categories (category (1)—reference groups (0)) and for Relative Income as continuous predictor with steps of 25% relative income increase

*P* value: * <0.050; ** <0.010, *** <0.001

## Discussion

In this study, we explored the impact of several gender dimensions on the health technology self-efficacy (HTSE) and intention for uptake of digital health technologies (DHT) among men and women with PD. We found that gender role orientation was a stronger predictor of HTSE and intention to use DHT compared to the dimension of gender identity and gender relations. Particularly, an androgynous gender role orientation was associated with higher general self-efficacy (GSE), health technology self-efficacy (HTSE) and more positive attitude toward health technology (HTA), meaning that men and women with PD who endorse both Western stereotypical masculine *and* feminine (non-binary) gender role orientations, as opposed to those that orient toward *either* a masculine *or* feminine (binary) gender role orientation, are therefore more likely to adopt health technologies to monitor their health and treat their medical condition.

The impact of gender-related roles and norms on health inequalities have been emphasized in previous publications [28–32]. Disentangling dimensions of gender identity and normative gender roles enables a more complex investigation into the influence of gender-related norms, roles and stereotypes in access to and interactions with health technology for people with PD of similar and different socio-demographical backgrounds. This approach to gender in relation to digital healthcare technology uptake and use focuses on the way how people are culturally ‘doing gender’ (gender as a behavioral construct) in relation to health technology, rather than assuming gendered patterns of health technology use among people with PD solely based on their gender identity, which can lead to false negative findings [33, 34]. The findings of our study endorse the calls of previous papers that emphasized the critical need for considering more behavioral and relational focused gender-sensitive analyses when examining differences and similarities between women, men and non-binary people in the context of (non)digital health technology use [16, 35–38]. We therefore highlight the need to move beyond the simplistic gender identity binary in gender-sensitive analyses of health technology evaluations.

The relationship between gender roles, norms and relations and health technology is highly contextual and can be influenced by intersecting social demographics, such as education type, relative income, sexual orientation, (dis)ability, generation/age, social class/caste, (multi)ethnicity and racialization [1, 37]. Gendered experiences in self-efficacy, attitude toward and uptake of health technologies can therefore vary significantly between and within gender identity categories when multiple intersecting social demographics or clinical outcomes are considered. Therefore, we showcased a novel exploratory intersectional gender analysis to examine how gender identity, roles and relations, intersect with education, employment, disease duration and disease severity and affect health technology self-efficacy (HTSE) and health technology attitude (HTA) among people with PD. To our knowledge, this is a first intersectional gender analysis applied in the context of health technology and PD. We emphasize that the sample sizes for this exploratory analysis may not have provided adequate statistical power for robust intersectional conclusions and that the results should therefore be interpreted with caution. We accentuate the exploratory intention for this analysis to demonstrate an intersectional gender approach that could be replicable in more detailed (case) studies with more extensive study populations.

Our exploratory intersectional gender analysis suggested that an androgynous gender role, compounded with higher education, unemployment, or lower disease severity appeared to be related to better HTSE, whereas an undifferentiated gender role, compounded with a lower education level, unemployment, and relatively higher disease duration predicted related to worse HTSE. Additionally, an androgynous gender role, compounded by higher education, unemployment, or a relatively shorter disease duration, predicted more positive HTA, whereas an undifferentiated gender role, compounded with a lower education level, unemployment, a relatively shorter disease duration, or more severe disease severity predicted more negative HTA. These results indicate, cautiously, that the impact of normative gender roles on HTSE and HTA can be moderated by these sociodemographic and clinical factors and are therefore potentially relevant to consider in conjunction with gender dimensions through intersectional analyses. The observed effect sizes for HTSE and HTA ranged from small to medium, with some reaching medium to large, highlighting the importance of considering the nuances and contexts of these effects in further research and practical applications.

Digital health technologies are often presented as having the potential to boost efficiency, increase personalization of PD care and provide more equitable access to PD healthcare services [1, 39]. However, DHT that are supposed to lessen disparities can also potentially exacerbate them for particular sub-populations of people with PD when social determinants, such as gender, are looked at as single factors rather than as factors that are moderated in the presence or absence of other intersecting social or disease-specific factors, resulting in compounded advantages or disadvantages in uptake and use of these DHT [40]. Intersectionality theory applied to DHT suggests that each individual’s identity and lived experience is multifaceted and that individuals will use (or will not use) digital health services based on their own unique circumstances, rather than as members of a single social category (Man/Woman, Higher/Lower Educated, Younger/Older, Higher/Lower Disease Severity, etc.). With a growing interest in assessing and monitoring PD symptoms with digital and mobile health technologies, it is simultaneously important to consider these intersectional factors in detailed case studies to better understand how different aspects of (dis)advantages interact with individual lives of people with PD. Ultimately, this should support more successful adoption of health technologies into PD care while reduce the risk of, unintentionally, causing or increasing digital health disparities between different social groups of men and women with PD [39, 40].

### Methodological limitations and considerations

To capture multiple gender dimensions in our survey, we used a gender assessment including state-of-the-art options for operationalizing the gender dimensions as applied to this study. Currently, there is no golden standard for the operationalization of these gender dimensions in medical research. Therefore, it remains critical to clearly define the underlying classifications to support reproducibility. Although the BSRI is still widely used as a measure for gender roles, it has encountered valid criticism regarding the categorization of masculine and feminine traits and the cultural and time sensitivity of gender stereotypes [41, 42]. Furthermore, we operationalized gender relations through involvement in household tasks and relative income using self-reported responses from the participants only. For more robust data interpretation, the recording of the responses of both the participants and their partners/spouses to these items would be preferred.

Also, the HTSE questionnaire was not specifically designed to detect HTSE among people with PD. While using a generic HTSE measure allows for comparison between individuals with PD and people with other chronic diseases, disease-specific factors may affect the ease and confidence with which people with PD use health technologies. Our exploratory intersectional gender analysis showed that disease-specific factors, such as disease duration and disease severity, can potentially affect HTSE and HTA but only on the behavioral dimension of gender role orientation, indicating their intersection with social gender norms and stereotypical roles.

The finding that individuals with androgynous gender roles exhibited better HTSE and HTA was based on our initial hypothesis, thus rejecting the null hypothesis. Specifically, androgynous gender roles were associated with higher HTSE and more positive attitudes toward health technology with medium effect sizes. This effect size, while indicative of meaningful associations, also highlight a limitation in terms of the strength, emphasizing the need for further research with larger sample sizes to confirm these findings.

We also reported preliminary findings of our intersectional gender analysis intended for replication due to limitations on statistical power. Replicating these results in future studies is essential to validate the observed trends and to further investigate the complex interactions between gender roles, social factors, and disease-specific factors in influencing HTSE and HTA.

## Conclusion

This study underscores the importance of viewing gender as a multidimensional social variable rather than focusing solely on gender identities when examining health technology adoption in PD research. By considering gender roles, we observe more significant associations with HTSE and HTA compared to other gender identity and relations, although effect sizes were generally small. Additionally, the impact of gender dimensions on HTSE and HTA can be compounded by intersecting social and disease-specific factors, potentially worsening or improving the intention of men and women with PD to use digital health technologies. Future studies should consider multiple gender dimensions alongside intersecting social and disease-specific factors to fully understand the compounded advantages or disadvantages in technology uptake and use among people with PD.

## Supplementary Information

Below is the link to the electronic supplementary material.

Supplementary file1 (DOCX 17 KB)

## Data Availability

Not applicable.
