# Supplementary material for: A multidimensional gender analysis of health technology self-efficacy among people with Parkinson’s disease
Source: J Neurol. 2024 Aug 22;271(10):6750–60. doi: 10.1007/s00415-024-12635-3 (PMC11457693; doi:10.1007/s00415-024-12635-3)
Supplement: Supplementary file 1 — Supplementary file1 (DOCX 17 KB) [file 415_2024_12635_MOESM1_ESM.docx]

**Supplement 1.** Overview of the included gender dimensions assessments

| **Dimension** | **Endpoint** | **Instrument** | **Reference** |
| --- | --- | --- | --- |
| Gender identity | Self-defined gender identity | Single Item Question | Lindqvist et al. 2020. “What Is Gender, Anyway: A Review of the Options for Operationalising Gender.” Psychology and Sexuality. |
|  | Sex assigned at birth | Single Item Question | Lindqvist et al. 2020. “What Is Gender, Anyway: A Review of the Options for Operationalising Gender.” Psychology and Sexuality. |
|  | Sexual orientation | Single Item Question | Lindqvist et al. 2020. “What Is Gender, Anyway: A Review of the Options for Operationalising Gender.” Psychology and Sexuality. |
| Gender Roles | Gender Expression | Single Item Question | Lindqvist et al. 2020. “What Is Gender, Anyway: A Review of the Options for Operationalising Gender.” Psychology and Sexuality. |
|  | Gender Role Orientation | Bem Sex Role Inventory | Bem, Sandra L. 1974. “The Measurement of Psychological Androgyny.” Journal of Consulting and Clinical Psychology. US: American Psychological Association. |
| Gender Relations | Private - Living Situation | Singe Item Question | Horne, et al. 2018. “Time, Money, or Gender? Predictors of the Division of Household Labour Across Life Stages.” Sex Roles |
|  | Private - Childcare | Single Item Question | Horne, et al. 2018. “Time, Money, or Gender? Predictors of the Division of Household Labour Across Life Stages.” Sex Roles |
|  | Private - Division of household labor | Seven Item Question | Horne, et al. 2018. “Time, Money, or Gender? Predictors of the Division of Household Labour Across Life Stages.” Sex Roles  Fernández, et al. 2016. “The Gendered Division of Housework.” Psicothema   Newkirk, et al. 2017. “Division of Household and Childcare Labor and Relationship Conflict Among Low-Income New Parents.” Sex Roles |
|  | Private - Relative income | Single Item Question | Horne, et al. 2018. “Time, Money, or Gender? Predictors of the Division of Household Labour Across Life Stages.” Sex Roles |
|  | Private – Paid and Unpaid labor | Single Item Question | Horne, et al. 2018. “Time, Money, or Gender? Predictors of the Division of Household Labour Across Life Stages.” Sex Roles |

**Author Information:**

Irene Göttgens1*, Sirwan K.L. Darweesh2, Bastiaan R. Bloem2, Sabine Oertelt-Prigione1,3*

1 Department of Primary and Community Care, Radboud Institute for Health Sciences, Radboud University Medical Center, Nijmegen, The Netherlands.

2 Radboud University Medical Center, Donders Institute for Brain, Cognition and Behavior, Department of Neurology, Center of Expertise for Parkinson & Movement Disorders, Nijmegen, The Netherlands.

3 AG 10 Sex- and Gender-sensitive Medicine, Medical Faculty OWL, University of Bielefeld, Bielefeld, Germany.

*Corresponding Author

Irene Göttgens, PhD, MSc

Radboud University Medical Center

Department of Primary and Community Care

Postbus 9101, 6500 HB Nijmegen

The Netherlands

Email: Irene.gottgens@radboudumc.nl
